# Biomaterials in Skin Wound Healing and Tissue Regenerations—An Overview

**DOI:** 10.3390/pharmaceutics14061291

**Published:** 2022-06-17

**Authors:** Marek Konop

**Affiliations:** Department of Experimental Physiology and Pathophysiology, Laboratory of Centre for Preclinical Research, Medical University of Warsaw, 02-091 Warszawa, Poland; marek.konop@wum.edu.pl

Wound healing is a complex biological process. Wounds are divided into acute and chronic. Wounds can arise after acute trauma, tissue destruction in the process of chronic inflammation, or as the result of chronic venous insufficiency or arterial ischemia. The process of healing is influenced by many pathological processes such as diabetes, immunosuppression, inappropriate immune response, venous stasis, ischemia, or bacterial infection. Effective wound treatment is still a big challenge for many specialists.

Currently available methods of wound dressing in many cases are insufficient. This has driven research and trials to generate novel wound dressings that improve healing time and efficacy and show biocompatibility with treated tissue. Several different materials such as keratin [1], silk [2], marine polysaccharides [3], and membranous scaffolds [4] have been studied. The various bioactive properties of these biomaterials, including antibacterial, anti-inflammatory, and hemostatic properties, thus promote and accelerate wound healing. Moreover, these biomaterials have higher biocompatibility and biodegradability compared to synthetic materials. This Special Issue explores different topics concerning recent signs of progress in the field of biomaterials and their application in the field of wound healing and tissue regeneration.

The development of safe and effective new biomaterials for the treatment of wounds, especially chronic non-healing wounds, is urgent. In recent years, promising results have been observed using multicomponent dressing based on the combination of biomaterials with stem cells, drugs, and nanoparticles. Silina et al. [5] examined the efficacy of utilizing biomaterials based on chitosan and cellulose supplemented with cerium dioxide nanoparticles, and mesenchymal stem cells and these two treatments in combination compared with control wounds in Wistar rats. The most effective remedy was a combination of cerium dioxide nanoparticles (PCCD) with mesenchymal stem cells (MSC). The treatments in the PCCD and MSC groups were more effective than in the injections of a deproteinized hemoderivative of calf blood (DHCB) and ointment Dioxomethyltetrahydropyrimidine + Chloramphenicol (DCh) groups. Invasive drugs and DCh slowed the regeneration process. DHCB did not affect the rate of healing for acute wounds without ischemia during the first week. However, the proven efficacy of developed biomaterials needs further studies in clinical practice settings.

Alhakamy et al. [6] examine the formulation of ceftriaxone and melittin as nanoconjugate (nanocomplex)-loaded hydroxypropyl methylcellulose-based hydrogels for the healing of acute wounds in diabetic rats. They also used commercially available ointment MeboTM as a control. They showed that the CTX–MEL nanocomplex significantly increases tissue levels of VEGF-A and TGF-β1 and can improve wound healing in diabetic rats.

Kim et al. [7] examined a complex coacervate system consisting of high or low molecular (HW or LW) weight gelatin Type A (GA) and sodium alginate (SA) for the delivery of fibroblast growth factor (bFGF). The HWGA-SA coacervate system was more effective in protecting bFGF from trypsin digestion than LWGA-SA. Moreover, bFGF complex coacervates enhanced the viability, collagen synthesis, and migration of HDF cells under hyperglycemic conditions in vitro. The HWGA-SA coacervate system has potential as a novel therapeutic modality for diabetic foot ulcers. However, future studies are needed for clinical translation of the obtained results in the form of in vivo studies in a diabetic wound model and storage stability tests.

Ceresa and co-workers [8] examined microfragmented adipose tissue (lipoaspirate), obtained with Lipogems^®^ technology as a pro-healing agent in vitro. They showed that the in vitro lipoaspirate possesses a paracrine effect on fibroblasts and keratinocytes proliferation, and migration and showed an antibacterial effect. In summary, the obtained results suggest that lipoaspirate may be a promising approach in wound healing, showing regenerative and antibacterial activities in vitro. Antibacterial and biofilm-preventing properties in the area of wound healing are necessary. Borgolte et al. [9] prepared a polymeric design of polymer-bound *N*-acetyl-glucosamine-oligoethylene glycol residues that mimic cationic, antibacterial, and biocompatible chitosan surfaces. Different polymers containing an *N*-acetylglucosamine-methacryloyl residue with oligoethylene glycol linkers and a methacryloyl benzophenone crosslinker were synthesized by the free radical polymerization method. The obtained polymers showed no cytotoxic or antiadhesive effects on fibroblasts cell culture methods. Moreover, biofilm formation was reduced by up to 70% and antibacterial growth by 1.2 log, particularly for the 5% GlcNAc-4EG polymer, as observed for *E. coli* and *S. aureus*.

The excessive production of reactive oxygen species (ROS) causes harmful effects, including biomolecular damage, increased inflammatory reaction, and delayed wound healing. In this context, looking for stable antioxidants that can relieve excessive oxidative stress in the injured tissues, Yang and co-workers [10] obtained and examined idebenone-nanoparticles (IB@NPs) with high and stable antioxidant activity for efficient wound healing in vitro. IB@NPs showed excellent biocompatibility, inhibited oxidative damage to mouse NIH-3T3 fibroblasts, and reduced intracellular ROS generation according to an in vitro DPPH antioxidant assay. Furthermore, synthesized IB@NPs significantly accelerated wound healing in a scratch assay model.

Domaszewska-Szostak et al. [11] developed a method for the long-term preservation of human skin for isolation and effective keratinocyte (KCs) transplantation to wounds. The originality of the proposed method consists of an effective storage procedure and an easy method of KCs preparation for transplantation. The preservation in anhydrous, pulverized sodium chloride inhibits KCs proliferation but did not stop some cells from displaying enzymatic activity upon setting in culture. An in vivo experiment showed that transplantation to SCID mice brought about the restarting of mitoses. Moreover, KCs transplanted to the wound area provide a physiological barrier and may also be a source of proliferating transient cells and cytokines regulating the growth of their own and host keratinocytes and fibroblasts.

Nowak et al. [12] examined the effect of advanced platelet-rich fibrin during the surgical extraction of third molars on healing and the concentration of c-reactive protein. They showed that the concentration of reactive protein C in the peripheral blood, 7 days after the surgical extraction of the impacted tooth, was lower in patients who received A-PRF blood product intra-operatively. Moreover, they showed that even slight inflammation associated with the difficult eruption of third wisdom teeth causes a slight increase in CRP protein. The A-PRF therapy allows a faster reduction in CRP concentration after the procedure and significantly reduces the incidence of postoperative complications in the form of a dry socket.

This Special Issue was completed by five reviews providing an overall view of the use of various biomaterials in tissue regeneration. In particular, Konop et al. [1] described the biomedical properties of keratin biomaterials in clinical and preclinical studies. They showed that soluble or insoluble keratin biomaterials are safe, non-toxic, and accelerate wound healing in healthy and diabetic conditions. Another study focuses on the different applications of silk-fibroin-based biomaterials. Lehmann et al. [2] described different forms of silk fibroin, including nanoparticles, biosensors, tissue scaffolds, wound dressings, and novel drug-delivery systems. They showed that silk fibroin can be combined with other biomaterials, such as chitosan or microRNA-bound cerium oxide nanoparticles (CNP), to have a synergistic effect on improving impaired wound healing. Shen and co-workers [3] described the properties of various marine polysaccharides including marine animals, algae, and microbial polysaccharides. These biomaterials have higher biocompatibility and biodegradability compared to synthetic ones and showed antibacterial, anti-inflammatory, and hemostatic properties. Moreover, marine polysaccharides can be combined with copolymers and active substances to prepare various forms of dressings such as nanofibers, smart hydrogels, and injectable hydrogels.

Da et al. [4] described the possibility of the application of extracellular matrix (ECM) scaffolds obtained from different types of body membranes. Three-dimensional (3D) printing technology can be used for the preparation of membranous ECM-based scaffolds. This technique can be used for the fabrication of skin grafts with structures closer to natural skin. In vivo results showed that the printed multilayer skin equivalent accelerated wound healing, as seen by improved re-epithelization, dermal ECM secretion, and angiogenesis

In the last review, Lombardo et al. [13] analyze possible therapeutic and surgical options that go beyond traditional meniscal surgery: from scaffolds, which are made of different kinds of polymers, such as natural, synthetic, or hydrogel components, and 3D printing construct or hybrid biomaterials made of scaffolds and specific cells.

In summary, the most important challenges in the upcoming decades will be to find new biomaterials that will accelerate the wound healing process, especially in pathological conditions such as diabetes, and to translate them from the laboratory to clinical trials. However, to overcome this problem, innovative solutions from interdisciplinary teams composed of chemists, bioengineers, and medical doctors, as well as regulatory policies that facilitate access to research and patients, are necessary.
